# Innovative Use of a Pediatric Double-Lumen Central Venous Catheter as a Midline Substitute in Adults With Difficult Venous Access: A Report of Two Cases

**DOI:** 10.7759/cureus.97014

**Published:** 2025-11-16

**Authors:** Tushar Mantri, Jyoti Burad, Shilpa Ramachandran

**Affiliations:** 1 Anesthesia and Intensive Care, Sultan Qaboos University Hospital, Muscat, OMN; 2 Emergency Medicine, Sultan Qaboos University Hospital, Muscat, OMN

**Keywords:** difficult venous access, innovative strategies, midline catheter, paediatric central venous catheter, ultrasound-guided cannulation

## Abstract

Long-term venous access is a dictum in recently advanced therapeutics, offering innumerable treatment options. When the patients are critically sick, central lines provide access for inotropes, antibiotics, dialysis, and hyperoncotic medications. However, when the patients step down to the intermediate level, only a few safe options of venous access are left. Midline catheters are intermediate-length venous access devices (3-5 French, 8-25 cm) inserted into the deep upper extremity veins, such as the basilic or cephalic vein, to provide reliable access for patients requiring prolonged therapy. As it terminates in the peripheral venous system, midlines offer a safer alternative to central venous catheters (CVCs), minimizing risks of pneumothorax, thrombosis, and infection. Midline catheters provide durable peripheral venous access but may be unavailable in some hospitals. Pediatric CVCs are typically 3-5 French and 5-10 cm in length and share dimensional similarities with midline catheters, suggesting potential for adaptation in adults when standard options are lacking. These devices are widely available and can be a substitute when standard devices are unavailable. This strategy offers certain advantages, particularly in patients with difficult venous access who require more durable or higher-capacity intravenous (IV) therapy than what peripheral IVs can provide. We report two adults with difficult venous access, one with a neuromuscular disorder and hypercapnic respiratory failure and one with immune-mediated necrotizing myopathy and multiple prior central devices, in whom a 4-French (Fr), 8 cm, double-lumen pediatric CVC (Arrow^R ^CS-25402, Teleflex Medical Wayne, PA, USA) was inserted as a midline. Under real-time ultrasound guidance, the basilic vein was cannulated using a modified Seldinger technique, and the catheter was advanced with the tip positioned in the proximal basilic vein, distal to the axillary vein, consistent with midline standards. Both devices functioned without thrombosis, dislodgement, or catheter-related infection during the required intensive care unit (ICU) therapy. When patients require long-term stay, are hemodynamically stable, and require prolonged venous cannulation and standard midlines are unavailable, a size-matched pediatric CVC placed under ultrasound with a confirmed peripheral tip can provide safe, practical access. This approach offers a practical solution for many complex patients across diverse healthcare settings, potentially broadening venous access options where midline catheters are not routinely stocked. This off-label strategy should follow infection-prevention bundles, non-vesicant infusion limits, clear labeling, and documentation. Both single- and double-lumen pediatric CVCs may be considered where dimensional compatibility with midline specifications is ensured. Larger studies are warranted.

## Introduction

Patients who survive critical illness often need intravenous (IV) access beyond the first few days for antibiotics, anticoagulation, diuretics, electrolyte correction, and symptom control, as well as frequent blood draws. In many of these patients, easy and obvious veins may not be accessible. Edema, scarring from repeated cannulation, and fragile vessels make short cannulas fail quickly. Re‑siting them is painful, time‑consuming, and inefficient. On the other hand, a central line exposes patients to central line-associated bloodstream infections (CLABSIs) and thrombosis that can prolong ventilation and length of stay and worsen outcomes. Choosing the least invasive device that can safely deliver the therapy is therefore a central principle of vascular access [1,2].

Midlines are intermediate‑length catheters placed in deep upper‑arm veins with the tip in the peripheral venous system, not the central circulation. That means much lower risks of pneumothorax and CLABSI than central‑tip devices, with dwell times measured in days to weeks when maintained properly. Guidance such as Michigan Appropriateness Guide for Intravenous Catheters (MAGIC) and European recommendations supports midlines for non‑vesicant therapy of intermediate duration and emphasizes ultrasound to improve first‑pass success and safety [1,3,4]. However, many hospitals, especially smaller or resource‑constrained centers, do not routinely stock midline kits. Pediatric central venous catheters (CVCs), however, are almost always available. Conveniently, the most common pediatric sizes (3-5 Fr, 5-10 cm) overlap with the dimensions of short midlines. If inserted under ultrasound into a large peripheral vein (ideally the basilic) and kept distal to the axillary vein, a pediatric CVC can serve as a peripheral‑terminating substitute for non‑vesicant therapy. This idea is consistent with selecting the least invasive device that still meets the goal of treatment [1,3-5]. It is important to clearly document the use of pediatric CVC as a midline in the medical records, and this will help infection surveillance, billing, and compliance with CLABSI reporting metrics.

This report offers an important solution to a common problem of continued and safe intravascular access for patients not so sick but who have difficult vascular access, thereby requiring longer use of a CVC or a midline, which is not readily available at many institutes.

## Case presentation

Here, we present two adults with difficult IV access in whom a 4 Fr, 8 cm double-lumen pediatric central venous catheterization set (Arrow^R ^CS-25402, Teleflex Medical Wayne, PA, USA) was used as a midline substitute. We describe the sonographic mapping, insertion technique, safeguards to ensure a peripheral tip, and clinical course. We then discuss where this strategy fits in care pathways, its trade‑offs, and how teams can implement it safely [1-4,5]. By leveraging the widespread availability of paediatric CVCs and ultrasound-guided technique, this approach provided safe and effective venous access in the intensive care unit (ICU). The case highlights a practical solution for patients with complex venous access needs, demonstrating that paediatric CVCs can serve as viable midline alternatives in settings where standard midlines are not readily accessible, potentially informing clinical practice across diverse healthcare environments. Ethical approval is exempted for case reports at our institute; however, written consent has been obtained from both patients.

Case 1

**Figure 1:**
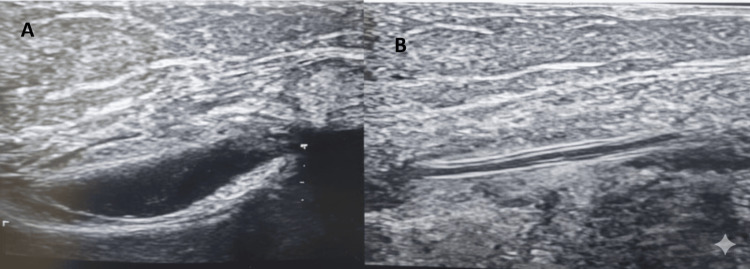
A: Guidewire inserted in the basilic vein. B: Pediatric central line in the basilic vein.

A 42-year-old male with a longstanding neuromuscular disorder presented to the emergency department with acute respiratory distress. Diagnosed with type 2 respiratory failure, he was transferred to the ICU and stabilized on non-invasive ventilation (NIV) with high settings (FiO_2_ 60%, IPAP 20 cmH_2_O, EPAP 8 cmH_2_O). His vital signs included a heart rate of 118 beats/min, blood pressure of 108/78 mmHg, respiratory rate of 55 breaths/min, and oxygen saturation of 98%. The patient’s chronic inability to lie supine, combined with significant dependent edema in the lower extremities and contractures, posed substantial challenges for vascular access. Multiple attempts at peripheral IV cannulation were unsuccessful, necessitating an alternative approach for blood sampling and medication administration. Central venous catheterization was contraindicated due to the patient’s positional limitations and risk of complications in the presence of edema. As a standard midline catheter was unavailable, a 4-French, 8 cm, double-lumen paediatric CVC was selected as an innovative alternative. Paediatric CVCs are widely available in most hospitals for paediatric and critical care use, and their dimensions (3-5 Fr, 5-10 cm) align closely with those of midline catheters (3-5 Fr, 8-25 cm), making them suitable for midline placement. The basilic vein of the right upper extremity was chosen for its large diameter (approximately 5 mm) and straight path, identified via ultrasound. The procedure was performed using a modified Seldinger technique under real-time ultrasound guidance. An 18-gauge cannula was inserted into the basilic vein at the mid-arm level, approximately 12 cm distal to the axilla. After needle removal, a guidewire was threaded as shown in Figure 1A, and the 4-French catheter was advanced to its full 8 cm length (Figure 1B), positioning the tip in the proximal basilic vein, approximately 4-5 cm distal to the axillary vein, consistent with midline catheter placement. 

**Figure 2:**
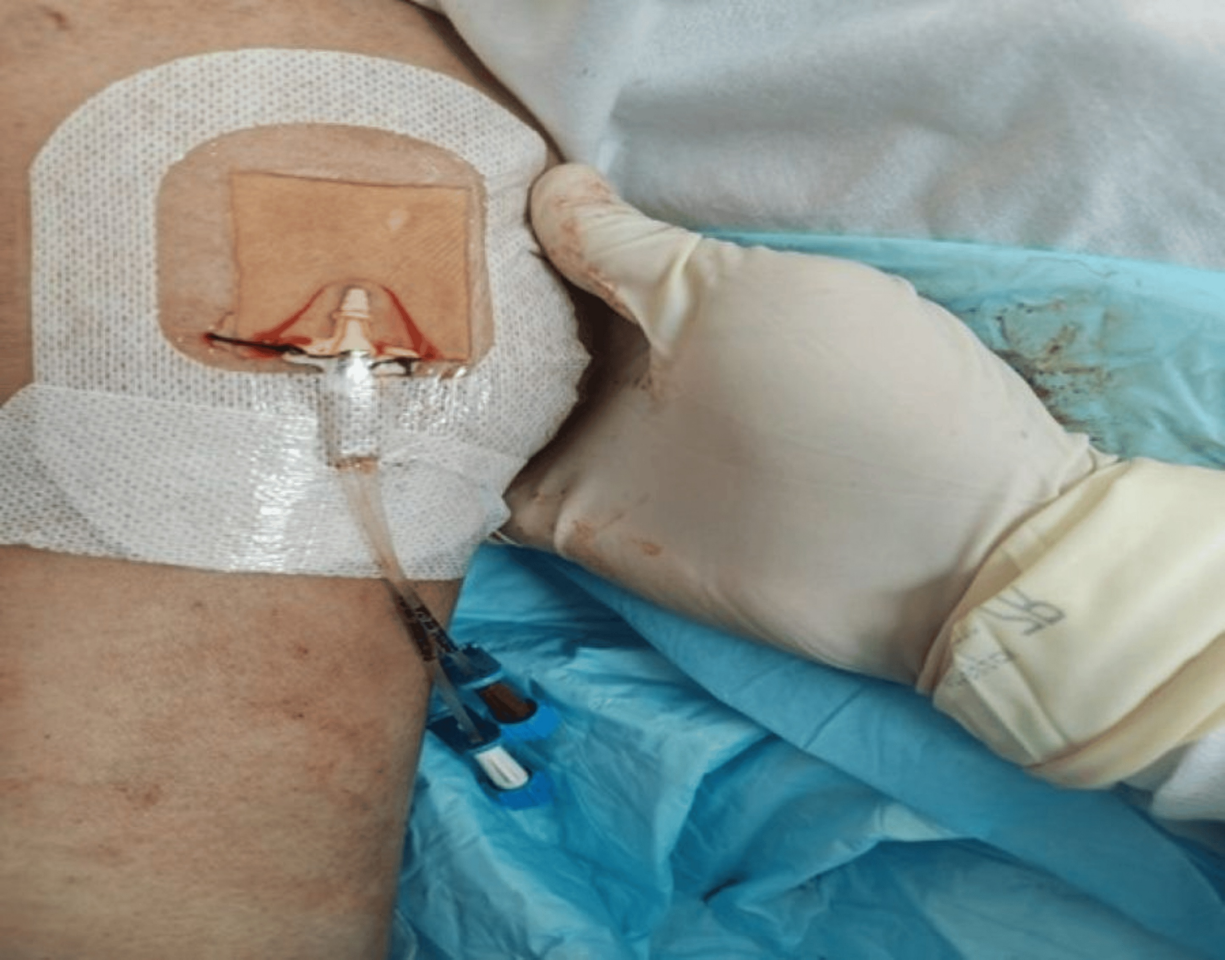
Secured two-lumen pediatric central line in the basilic vein

Ultrasound confirmed the peripheral tip position, ensuring no central venous entry. The catheter was secured and flushed to verify patency (Figure 2).

Case 2

**Figure 3:**
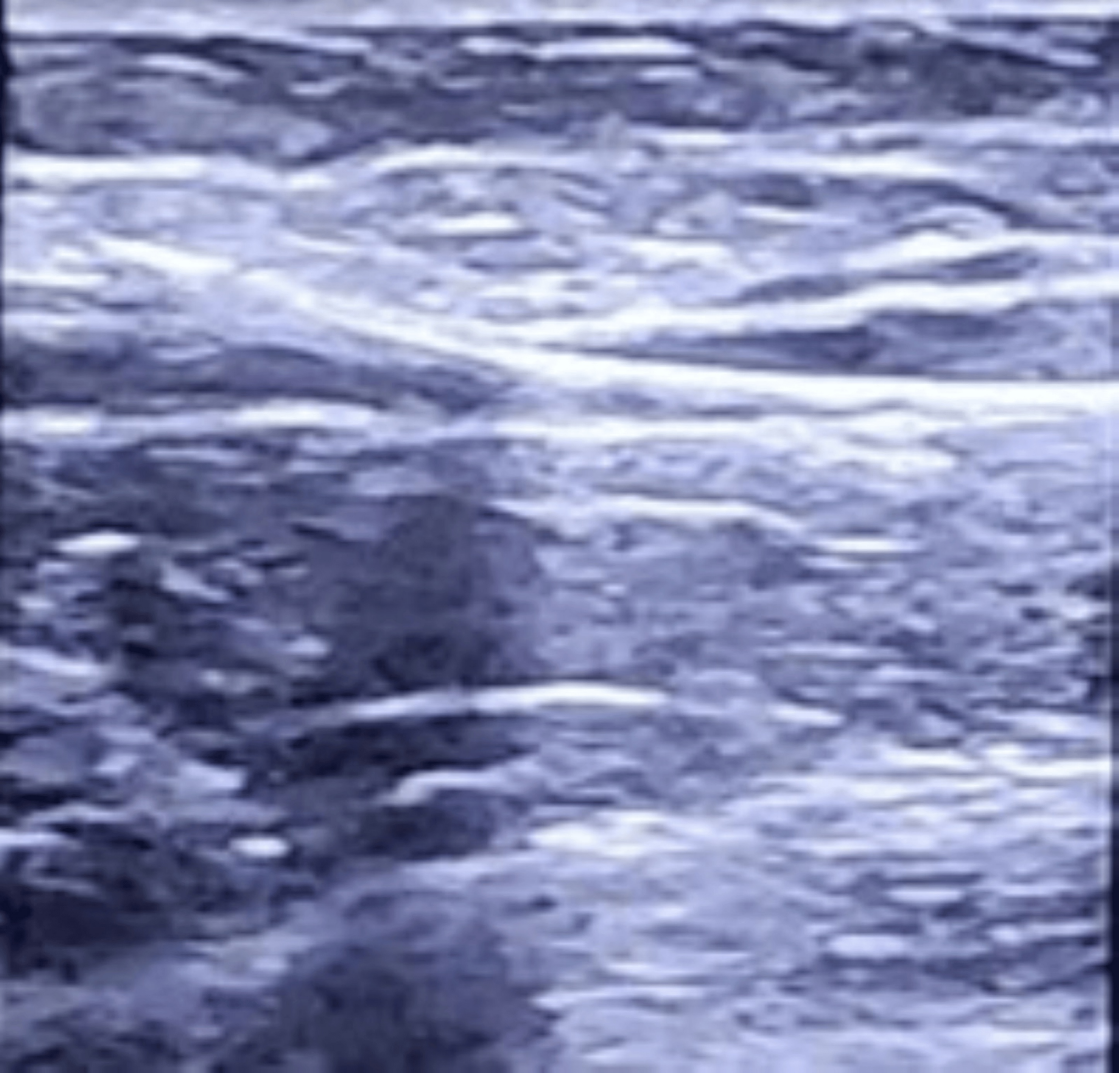
Guidewire in the basilic vein

The second case was a 44-year-old patient with proximal muscle weakness due to immune-mediated severe necrotising myopathy. She had a prolonged ICU course of more than a month during which she received plasmapheresis, IV immunoglobulin, and pulsed steroids. She developed line-related bacteremia and ventilator-associated pneumonia. She also had a uterine mass, acute kidney disease requiring dialysis, and myocarditis. Her central veins were used up for multiple catheters for dialysis, central venous access, and plasmapheresis. She was conscious with limited muscle power, breathing through a tracheostomy. She had normal vital signs and was not on vasopressors. There was a need for continued IV access; however, the central veins were already in use with a CVC and a dialysis catheter at the time and had been previously as well. We wanted to insert a peripheral cannula; however, the veins on the hands and feet were already thrombosed, and the limbs were also edematous. A need for insertion of a mid-size venous catheter was realized. However, midlines were out of stock, and hence, a decision was taken to insert a pediatric CVC in the mid-size basilic vein (diameter 0.25 cm). An 18-gauge cannula was inserted in the basilic vein at the distal arm level 5 cm above the elbow under ultrasound guidance. The stilette was removed, and the guidewire of the pediatric central line (4 French with an outer diameter of 1.5 mm) was threaded through the sheath of the cannula (Figure 3).

**Figure 4:**
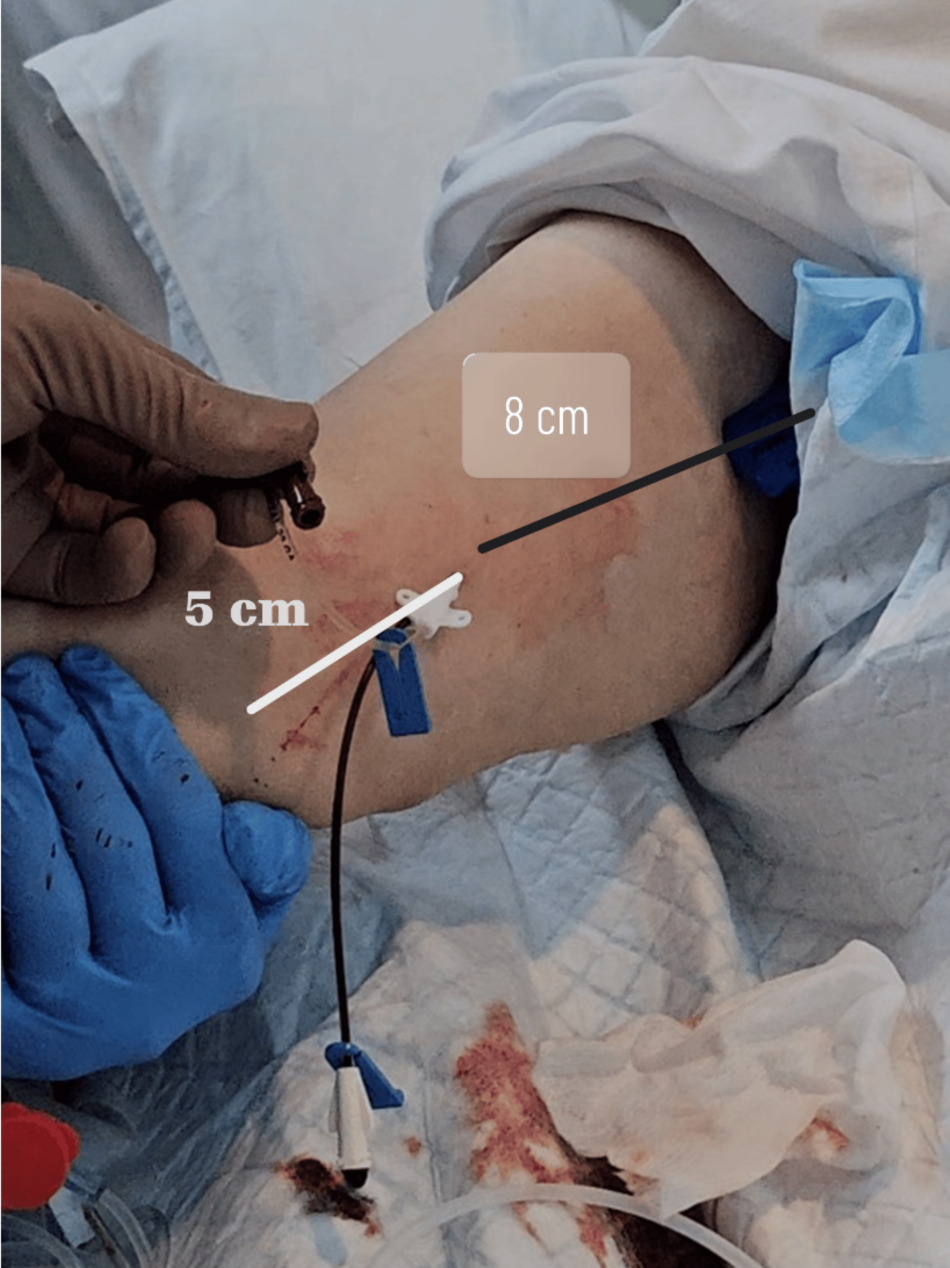
Insertion site of pediatric central venous catheter (CVC): 5 cm proximal to the elbow, extending 8 cm onto the arm.

The peripheral venous catheter was removed over the guidewire, and the pediatric central line was inserted over it for a full length of 8 cm (Figure 4).

The tip of the line was confirmed in the basilic vein, and the site was dressed with a sterile dressing used for central venous cannulation. The double-lumen design facilitated the simultaneous administration of antibiotics, fluids, and peripheral total parenteral nutrition (TPN), which are critical for the patient’s ICU management. No complications, such as thrombosis, infection, or catheter dislodgement, were observed during the patient’s ICU stay.

Diagnostic assessment

Tests and Imaging

Point-of-care ultrasonography (POCUS) of the upper limbs was used to map target veins before cannulation. For Case 1, the right basilic vein measured approximately 5 mm in diameter at mid-arm, with adequate depth, compressibility, and color-flow Doppler confirming patency and absence of intraluminal thrombus. For Case 2, the distal basilic vein measured approximately 2.5 mm and was patent despite limb edema; superficial hand and forearm veins appeared thrombosed. Dynamic ultrasound with distal augmentation confirmed flow, and the axillary confluence was identified to ensure the selected 8 cm catheter would terminate in the proximal basilic vein, distal to the axillary vein. A bedside ultrasound was also used during insertion for real-time needle tracking and post-placement confirmation of a peripheral (non-central) tip location, consistent with guidance on ultrasound-guided vascular access [3].

Diagnostic Reasoning and Alternatives Considered

In both cases, standard peripheral IV cannulation had failed after multiple attempts, and clinical features (edema, contracted or immobile limbs, prior vein injury) predicted difficult access [2]. Non-tunneled CVC or PICC placement was considered but deprioritized due to (i) inability to tolerate supine positioning with high NIV support (Case 1), (ii) extensive concurrent central access requirements and recent line-associated bacteremia (Case 2), and (iii) the higher risks and resource demands of central access compared with a peripheral-terminating device when infusates were non-vesicant [1,4,5]. Intraosseous access was deemed unnecessary given hemodynamic stability and the need for intermediate-term therapy rather than emergent rescue. Long peripheral cannulas were considered unlikely to achieve acceptable dwell time or reliable flow in edematous, thrombosed superficial veins.

Challenges and How They Were Addressed

Key challenges included (1) inability to lie supine (Case 1), (2) generalized limb edema with thrombosed superficial veins (Case 2), and (3) risk of arterial or neural injury if the brachial vein were chosen. These were mitigated by (a) performing all mapping and cannulation under ultrasound while the patient remained on NIV in a semi-upright position, (b) selecting the basilic vein for its straight course, larger caliber, and distance from the brachial artery and median nerve, (c) using an in-plane approach for continuous needle-tip visualization, and (d) selecting a catheter length that ensured a peripheral tip, confirmed sonographically [3,4].

Prognostic Features and Risk Assessment

Pre-procedural ultrasound mapping demonstrated favorable vein caliber and trajectory, supporting a lower risk of insertion failure and early dysfunction. Choosing a peripheral-terminating device reduced the risk of CLABSI relative to a central-tip catheter while meeting therapy needs for non-vesicant infusions [1,5]. The double-lumen configuration in Case 2 was justified by concurrent infusion requirements, with ongoing surveillance for local phlebitis or thrombosis and line-care bundles applied to minimize infectious risk [5]. Overall, the diagnostic synthesis favored an ultrasound-guided, basilic-vein, peripheral-terminating catheter; in the absence of stocked midlines, a 4 Fr pediatric CVC of 8 cm length provided an anatomically appropriate and operationally safer alternative [1,3-5].

Patient Perspectives

Case 1: “I could stay upright and breathe easily while they placed the line, and it worked without more needle attempts.”

Case 2: “After many failed cannulas, this line was comfortable and spared me more needle sticks.”

## Discussion

CVCs are indispensable in critical care but carry a substantial risk of CLABSI, which is linked to prolonged mechanical ventilation and ICU stay, higher costs, and worse survival. Infection risk rises with repeated line manipulations, concurrent devices, and longer dwell times - common in complex ICU patients. When prescribed infusates are non-vesicant and within peripheral osmolality limits, peripherally inserted, peripheral-terminating options, such as midlines or size-matched pediatric catheters used as midline substitutes, can reduce infectious and mechanical hazards by avoiding intrathoracic puncture and a central tip, while still providing durable access. Contemporary guidance recommends selecting the least invasive device that safely meets therapeutic requirements and using ultrasound to optimize insertion and maintenance, thereby minimizing complications and CLABSI exposure. In settings where standard midlines are unavailable, an ultrasound-guided pediatric CVC adapted as a midline offers a pragmatic pathway to uphold these principles without compromising care [1,4,5].

Midlines offer durable access with lower risks of pneumothorax and CLABSI compared with CVCs, and ultrasound guidance enhances safety and success [1,3,4]. Pediatric CVCs are ubiquitous and, in selected sizes and lengths, can approximate midline characteristics. Critical safeguards include (1) ultrasound-guided insertion; (2) deliberate positioning of the tip within the proximal peripheral vein, distal to the axillary vein; (3) securement and labeling to prevent misclassification as a central line; and (4) adherence to non-vesicant infusion limits. The basilic-brachial confluence near the axilla typically lies 10-15 cm from a mid-arm insertion site, making central entry unlikely with an 8 cm catheter when positioned deliberately in the proximal basilic vein.

Technical considerations differ from standard peripheral cannulas. Pediatric CVCs (silicone or polyurethane) have internal diameters that support higher flow than small-gauge peripheral cannulas and are mechanically stable, enabling several days of use when maintained aseptically. One of the key benefits of using a pediatric CVC in place of a midline catheter is its smaller diameter and shorter length compared to standard adult CVCs. This makes it more comfortable and less traumatic when placed in deep peripheral veins such as the basilic or brachial vein. A dual-lumen design can allow for the simultaneous administration of incompatible medications, which most midline catheters cannot accommodate. In addition, these catheters may be more readily available in emergency or resource-limited settings, allowing clinicians to provide more reliable IV access without delay. Pediatric CVCs also support higher flow rates than regular peripheral IV cannulas and offer greater durability for intermediate-term use, if inserted and maintained using proper sterile techniques, while peripheral IVs generally require replacement after 72-144 hours due to phlebitis or infiltration risk [5-7]. While this supports their functional suitability as midline substitutes, their use in a peripheral location remains off-label; infection-prevention bundles should be rigorously applied, and dwell time minimized to clinical need. Clear documentation as a peripheral midline substitute aids surveillance and billing alignment with CLABSI metrics.

Limitations

Despite these advantages, using a pediatric CVC as a midline substitute comes with significant limitations. First, these catheters are not designed for midline insertion and may lack features such as appropriate stiffness, tip shape, or securement devices optimized for peripheral access. This increases the risk of malposition, migration, or dislodgement. Second, there is a risk of misclassification; clinical staff may mistake the catheter for a central line and use it for therapies such as parenteral nutrition, vesicants, or central venous pressure monitoring, which are inappropriate and potentially harmful in this context. Furthermore, pediatric CVCs may lack antimicrobial properties or other design features that reduce the risk of infection, thereby increasing the likelihood of catheter-related infections when used off-label in peripheral sites. These limitations can be mitigated by explicit labeling as a peripheral-terminating device, staff education, and adherence to line-care bundles. 

Institutions can operationalize this approach with a brief protocol covering candidate selection (non‑vesicant therapy; difficult access; central access undesirable), required ultrasound skills, standardized labeling, and nursing education. Future studies should compare this strategy with purpose‑built midlines and with repeated short PIVs on dwell time, replacement rates, infiltration/extravasation, thrombosis, CLABSI, cost, and workflow in resource‑limited settings.

## Conclusions

When midlines are unavailable, a 4 Fr, 8 cm pediatric double‑lumen CVC placed under ultrasound into a large peripheral vein and kept distal to the axillary vein can serve as a dependable midline substitute. In these two adults, it delivered needed non‑vesicant therapy without complications. With careful selection, meticulous technique, explicit labeling, securement, and maintenance bundles, this is a practical and safe option that reduces central‑tip exposure. Larger studies should be conducted to define comparative effectiveness and safety.
